# Ultrafast Photochemical Reaction Dynamics of 3-Phenyl-1,4,2-dioxazol-5-one Revealed by Femtosecond Time-Resolved Infrared Spectroscopy

**DOI:** 10.3390/ijms27125563

**Published:** 2026-06-19

**Authors:** Seongbeom Jeon, Juhyang Shin, Seongchul Park, Hyeonwoong Bae, Jongwoo Son, Manho Lim

**Affiliations:** 1 Department of Chemistry, Chemistry Institute for Functional Materials, Pusan National University, Busan 46241, Republic of Korea; s_b.21@pusan.ac.kr (S.J.); dksvmflzk@gmail.com (J.S.); 2 Institute for Future Earth, Pusan National University, Busan 46241, Republic of Korea; papagenona@gmail.com; 3 Department of Chemical Engineering (BK21 FOUR Graduate Program), Dong-A University, Busan 49315, Republic of Korea; mybhw1297@gmail.com

**Keywords:** dioxazolone photodecomposition, nitrene, isocyanate, decarboxylation, solution-phase reaction dynamics, femtosecond time-resolved vibrational spectroscopy

## Abstract

Dioxazolones are important precursors for generating nitrenes (highly reactive intermediates widely used for carbon–nitrogen bond formation in organic synthesis) upon exposure to light or heat. The photochemical reaction dynamics of 3-phenyl-1,4,2-dioxazol-5-one in CHCl_3_ were investigated using femtosecond time-resolved infrared spectroscopy and electronic structure calculations. Photoexcitation at 267 nm rapidly populates an excited singlet state that serves as the key branching point for subsequent photophysical and photochemical processes. Transient infrared spectra reveal the formation of carbon dioxide, phenyl isocyanate, and singlet benzoyl nitrene through their characteristic vibrational features. Kinetic analysis shows that decarboxylation from the excited singlet state occurs with a time constant of 4.7 ± 1 ns, producing phenyl isocyanate and benzoyl nitrene with time constants of 8.1 ± 2 ns and 11 ± 3 ns, respectively. Competing relaxation pathways include internal conversion to the ground state (7.5 ± 2 ns) and intersystem crossing to the T_1_ state (25 ± 5 ns). The T_1_ state relaxes to the ground state (350 ± 30 ns) without contributing to product formation. These results demonstrate that both isocyanate and nitrene products originate from the S_1_ state and provide detailed mechanistic insight into the competing pathways governing dioxazolone photochemistry in solution.

## 1. Introduction

Nitrenes are highly reactive intermediates that play a central role in C–N bond formation and a wide range of photochemical processes [1,2]. Their reactivity is strongly governed by their electronic structure, particularly the interplay between singlet and triplet states, which dictates pathways such as insertion, rearrangement, and addition [2,3,4]. Understanding how these electronic and structural factors control reaction pathways remains a key objective in chemical reaction dynamics. Among nitrene precursors, acyl azides and related carbonyl-containing systems have served as prototypical platforms for mechanistic studies [5,6,7,8]. Their photochemical decomposition can yield either carbonyl nitrenes or isocyanates via Curtius-type rearrangement pathways [5,9]. Early mechanistic interpretations emphasized ground-state processes, but time-resolved spectroscopic studies have established that these reactions are driven primarily by excited-state dynamics [5,9,10,11,12]. In particular, ultrafast time-resolved infrared (TRIR) measurements have demonstrated that both nitrene and isocyanate products originate directly from the first singlet excited state (S_1_), identifying it as the key branching point controlling product formation [5,9]. These results highlight the central role of excited-state evolution and energy flow in determining reaction outcomes.

Carbonyl nitrenes exhibit electronic structures distinct from those of alkyl and aryl nitrenes. Although many nitrenes possess triplet ground states, carbonyl nitrenes often favor singlet configurations, which are stabilized by the interaction between the carbonyl oxygen lone pair and the vacant nitrogen p-orbital [13,14,15,16]. This electronic structure leads to characteristic nitrene reactivity, including rapid rearrangement to isocyanates and competing insertion reactions. TRIR techniques have provided direct evidence for singlet benzoyl nitrene and related species, revealing their vibrational signatures and reaction dynamics [5,9,12,13]. These observations underscore the importance of the coupling between electronic structure and ultrafast dynamics in determining reactivity.

Dioxazolones have recently emerged as versatile precursors for generating acyl nitrene equivalents under mild conditions [3,17,18]. Their stability and ability to release CO_2_ upon activation make them attractive alternatives to conventional azide-based systems, and they have been widely exploited in nitrene transfer reactions [4]. Despite this growing importance, the fundamental photochemical dynamics of dioxazolones in solution remain largely unexplored, particularly under direct photoexcitation conditions.

A key unresolved issue concerns the primary excited-state processes following the photoexcitation of dioxazolones. Because these molecules typically absorb the UV region through π–π* transitions localized on the aryl substituent, subsequent relaxation pathways, including internal conversion (IC), intersystem crossing (ISC), bond cleavage, and decarboxylation, can lead to multiple competing reaction channels. However, the timescales and branching mechanisms governing these processes remain unclear. Whether nitrene formation proceeds directly from excited states or via intermediate species, and how energy redistribution influences product selectivity, remain poorly established.

Ultrafast TRIR spectroscopy provides a powerful approach for addressing these questions by enabling the direct observation of structural evolution through vibrational signatures with subpicosecond time resolution [19,20]. By tracking transient species and their kinetics, this technique enables differentiation among competing pathways and identification of the key intermediates governing reaction dynamics. Previous applications to acyl azides and related systems have demonstrated its ability to correlate excited-state decay with product formation and to distinguish between concerted and stepwise mechanisms [5,9,10,12].

In this work, we investigated the ultrafast photochemical reaction dynamics of 3-phenyl-1,4,2-dioxazol-5-one in solution using femtosecond TRIR spectroscopy combined with electronic structure calculations. By monitoring the temporal evolution of vibrational spectra following UV excitation, we directly observed the formation of CO_2_, phenyl isocyanate, and singlet benzoyl nitrene, and quantitatively resolved their kinetics. The results demonstrate that both nitrene and isocyanate products originate from a common excited-state precursor, establishing the S_1_ state as the primary branching point governing product formation. These findings provide a detailed mechanistic framework for dioxazolone photochemistry and broader insight into the role of excited-state dynamics in controlling nitrene generation in solution.

## 2. Results and Discussion

**Figure 1:**
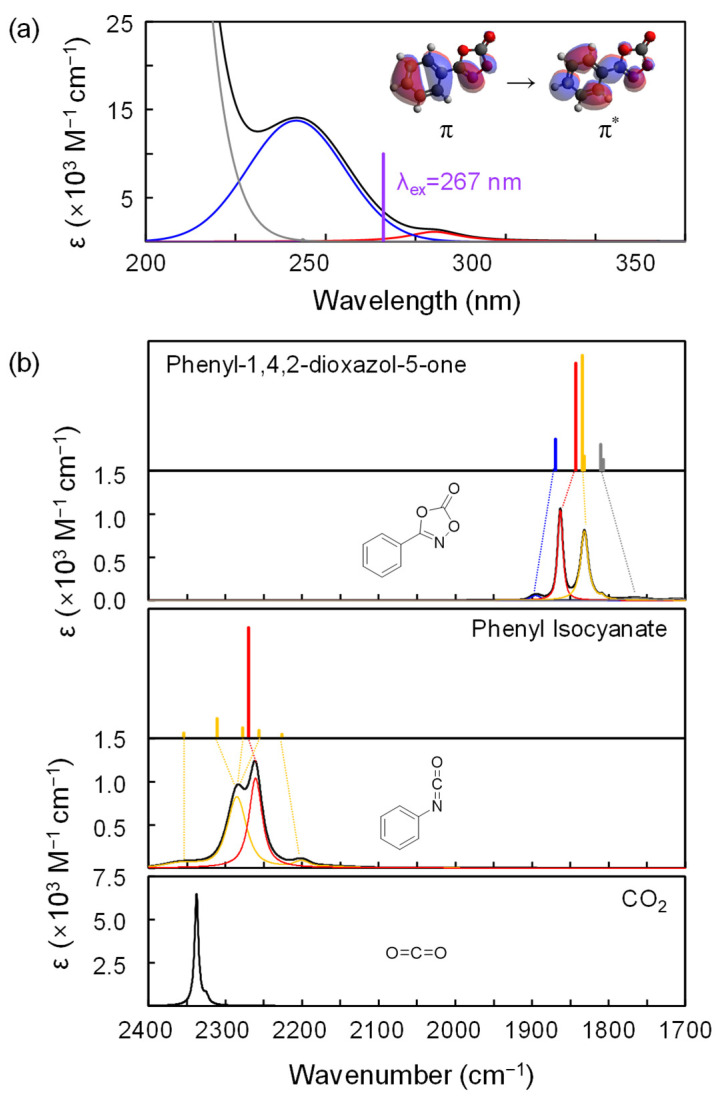
(**a**) Equilibrium ultraviolet–visible (UV–vis) absorption spectrum of 3-phenyl-1,4,2-dioxazol-5-one in CHCl_3_ at room temperature. The spectrum is decomposed into two bands centered at 243 nm (blue) and 281 nm (red), assigned to transitions to the S_2_ and S_1_ states, respectively (Figure S1 and Table S1). The intense 243 nm band corresponds to a π−π* transition localized on the phenyl group, as supported by time-dependent density functional theory (TD-DFT) calculations. The inset shows the molecular orbital associated with the S_2_ transition. The purple stick indicates the excitation wavelength, 267 nm, used for TRIR measurements. (**b**) Equilibrium Fourier transform infrared (FTIR) spectra of 3-phenyl-1,4,2-dioxazol-5-one, phenyl isocyanate, and CO_2_ in CHCl_3_ at room temperature. The experimental spectra (black lines) are decomposed into individual bands (red, orange, or blue lines), with assignments based on DFT calculations at the ωB97X-D/aug-cc-pVTZ level. The calculated vibrational spectra are shown as color-coded stick spectra. Note that the *y*-axis scale for the CO_2_ spectrum is five times larger than those used for the other spectra.

The electronic absorption spectrum of 3-phenyl-1,4,2-dioxazol-5-one exhibits two distinct absorption bands (Figure 1a). The intense band at 243 nm is assigned to a π–π* transition to the phenyl-localized S_2_ state, as supported by time-dependent density functional theory (TD-DFT) calculations at the ωB97X-D/aug-cc-pVTZ level (Table S1 and Figure S1) [21,22,23]. Excitation at 267 nm therefore predominantly populates the S_2_ state. The equilibrium Fourier transform infrared (FTIR) spectra of 3-phenyl-1,4,2-dioxazol-5-one, phenyl isocyanate, and CO_2_ in CHCl_3_ at room temperature are shown in Figure 1b and provide reference vibrational signatures for assigning transient species in the TRIR spectra. The characteristic bands of 3-phenyl-1,4,2-dioxazol-5-one are assigned, on the basis of DFT calculations at the ωB97X-D/aug-cc-pVTZ level (Tables S2 and S3), to the C=O stretching mode (ν_40_ in Figure S2) at 1863 cm^−1^, a Fermi resonance-enhanced combination band involving ν_12_ and ν_31_ (Figure S2) at 1832 cm^−1^, and an overtone of the C_2_NO_2_ ring-breathing mode (ν_20_ in Figure S2) at 1895 cm^−1^. Phenyl isocyanate exhibits two prominent bands at 2285 and 2261 cm^−1^. The band at 2285 cm^−1^ is attributed to a group of overlapping combination bands in this spectral region, including ν_10_ + ν_30_, ν_17_ + ν_24_, and ν_12_ + ν_28_ (Figure S3; Tables S4 and S5), which may be further enhanced through Fermi resonance. The band at 2261 cm^−1^ corresponds to the N=C=O asymmetric stretching mode (ν_31_ in Figure S3) [12,24,25]. As shown in Figure S4, the simulated spectra based on these assignments reproduce the experimental spectra of both 3-phenyl-1,4,2-dioxazol-5-one and phenyl isocyanate reasonably well. CO_2_ in CHCl_3_ displays an intense band at 2338 cm^−1^ arising from the asymmetric stretching vibration. These assignments provide the foundation for interpreting the TRIR spectra and identifying transient intermediates and photoproducts.

**Figure 2:**
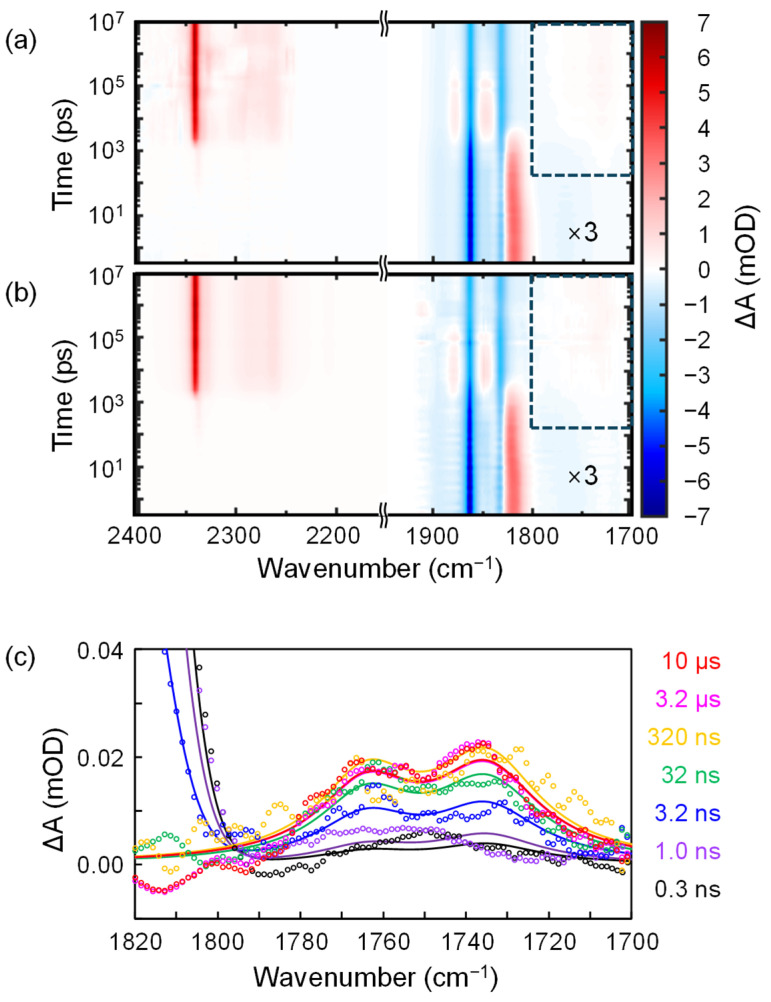
(**a**) Two-dimensional contour map of the femtosecond time-resolved infrared (TRIR) spectra of 3-phenyl-1,4,2-dioxazol-5-one in CHCl_3_ at 293 K following excitation at 267 nm. The 1820–1700 cm^−1^ region, which contains weak but diagnostically important features, is highlighted by a dashed box and expanded in panel (**c**). To facilitate visualization of weak transient features, the spectra in the 1950–1700 cm^−1^ region are scaled by a factor of 3. A version of the spectra with an expanded vertical scale is provided in Figure S5 to further enhance the visibility of these weak features. (**b**) TRIR spectra reconstructed from global fitting using the six basis spectra shown in Figure 3. (**c**) Expanded TRIR spectra (open circles) in the 1820–1700 cm^−1^ region at pump–probe delay times from 0.32 ns to 10 μs (color-coded), together with fits (solid lines) comprising two bands centered at 1764 and 1735 cm^−1^. These bands are assigned to singlet benzoyl nitrene based on literature values, 1765 and 1727 cm^−1^ in an Ar matrix [9], and DFT calculations. The pump-induced absorbance change (ΔA) was obtained by subtracting the unpumped spectrum from the pumped spectrum and is reported in optical density (OD; 1 mOD = 10^−3^ OD).

Femtosecond TRIR spectra were recorded over the spectral regions of 2400–2200 and 1950–1700 cm^−1^ across a time window of 0.3 ps to 10 μs following 267 nm excitation at 293 K (Figure 2a). The intermediate region, 2200–1950 cm^−1^, showed no significant transient features and was therefore omitted. Excitation at 267 nm, near the absorption edge, was chosen to minimize nonuniform excitation along the sample path length arising from the high optical density of the electronic transition relative to that of the probed mid-IR vibrational transitions. Such nonuniform excitation can lead to nonlinear absorbance responses [26]; excitation at 267 nm selectively populates the S_2_ state while mitigating these effects. Immediately after excitation (<0.3 ps), negative-going signals appeared at the ground-state vibrational band positions, indicating depletion of the reactant population. The prompt appearance of these bleach signals implies that ultrafast photophysical and/or photochemical processes occur within 0.3 ps.

**Figure 3:**
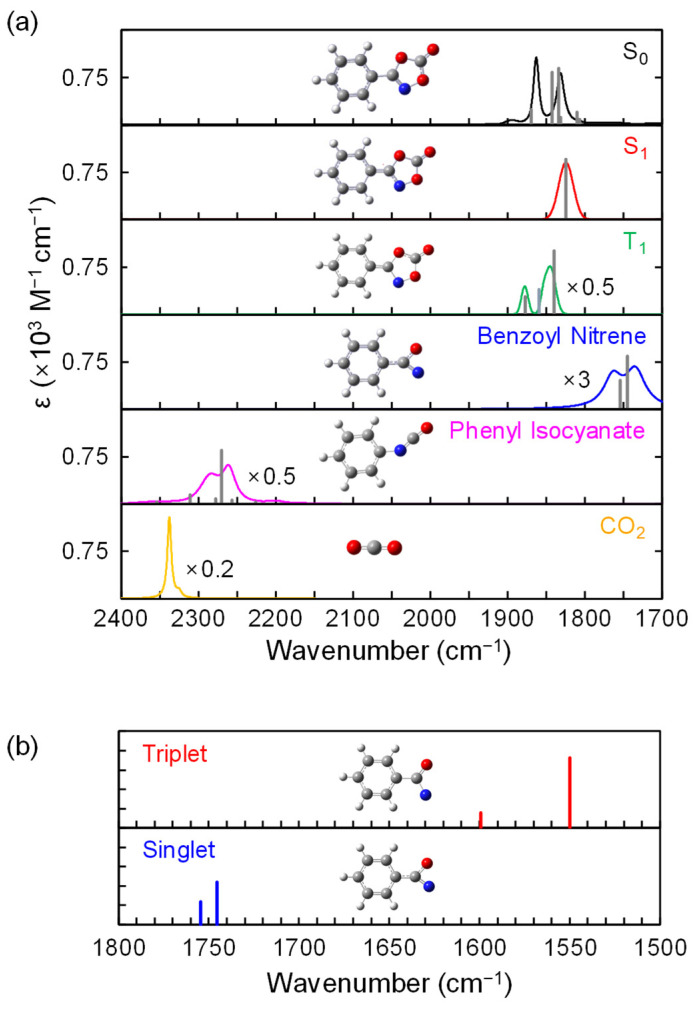
(**a**) Basis spectra used in the global analysis: ground-state reactant (S_0_, black), S_1_ (red), T_1_ (green), benzoyl nitrene (blue), phenyl isocyanate (pink), and CO_2_ (orange). Experimental spectra were used where available, whereas the excited-state spectra (S_1_ and T_1_) were derived from TD-DFT calculations (Tables S2 and S3) and further refined during fitting. The calculated vibrational spectra (gray sticks) were scaled by factors of 0.96–0.99 to match the experimental frequencies. For clarity, the T_1_, benzoyl nitrene, phenyl isocyanate, and CO_2_ spectra are scaled by factors of 0.5, 3, 0.5, and 0.2, respectively. (**b**) Optimized geometries and calculated vibrational spectra of benzoyl nitrene in the singlet and triplet states at the ωB97X-D/aug-cc-pVTZ level of theory (Tables S4 and S5). The triplet state exhibits a C=O stretching mode at 1550 cm^−1^, whereas the singlet state shows two characteristic bands at 1754 and 1745 cm^−1^, assigned to a combination mode of NCO rocking and phenyl ring Kekulé vibrations, and to the C=N stretching mode, respectively. The calculated vibrational frequencies (red or blue sticks) were scaled by a factor of 0.96 to match the experimental values.

The TRIR spectra reveal complex time-dependent evolution across multiple spectral regions. Key transient features include the following:

- A strong band at 1825 cm^−1^ that appears promptly and decays within a few nanoseconds.
- Two weak bands at 1878 and 1845 cm^−1^ that evolve over tens of nanoseconds.
- A strong band at 2338 cm^−1^ that appears within a few nanoseconds and persists.
- Two bands at 2285 and 2261 cm^−1^ that appear within a few nanoseconds and persist.
- Two weak but distinct bands at 1764 and 1735 cm^−1^ that emerge after 1 ns and persist.

The delayed appearance of the high-frequency bands at 2338, 2285, and 2261 cm^−1^ as well as the low-frequency bands at 1764 and 1735 cm^−1^ indicates that these features correspond to product formation rather than initial excited-state absorption.

The band at 2338 cm^−1^ is assigned to CO_2_, whereas the bands at 2285 and 2261 cm^−1^ are assigned to phenyl isocyanate, based on comparison with the equilibrium FTIR spectra and previous studies of decarboxylation reactions. Similar spectral features have been reported in photochemical studies of acyl azides and related carbonyl-containing systems. The weak bands at 1764 and 1735 cm^−1^ match the reported vibrational frequencies of singlet benzoyl nitrene absorptions, 1765 and 1727 cm^−1^ in an Ar matrix [9], and are therefore assigned to singlet benzoyl nitrene. This assignment is supported by TRIR studies of benzoyl azide photolysis and matrix-isolation experiments. Additional support comes from vibrational analysis based on DFT calculations, which reproduce these features as a combination mode of NCO rocking and phenyl ring Kekulé vibrations at 1764 cm^−1^ and the C=N stretching mode of the singlet nitrene at 1735 cm^−1^. In contrast, triplet nitrene species are expected to exhibit dominant absorptions near 1550 cm^−1^ (Figure 3b), which are not observed in the present spectra. The relative intensities and positions of the observed bands are consistent with singlet benzoyl nitrene, whereas the calculated spectra for the triplet state fail to reproduce these features. These results confirm the formation of singlet benzoyl nitrene upon photodecarboxylation of 3-phenyl-1,4,2-dioxazol-5-one. Importantly, the delayed growth of the nitrene bands indicates that nitrene formation proceeds through the evolution of an excited-state precursor. Similar behavior has been observed in ultrafast TRIR studies of acyl azides, in which nitrene formation is correlated with excited-state decay.

The appearance of product-related absorption features, including a band at 2338 cm^−1^, two bands at 2285 and 2261 cm^−1^, and two bands at 1764 and 1735 cm^−1^, indicates the formation of CO_2_, phenyl isocyanate, and benzoyl nitrene, respectively. The concurrent emergence of these species suggests a branching process originating from a common precursor. Previous studies of acyl azides have shown that nitrene and isocyanate formation can proceed through closely related excited-state pathways, with the branching ratio governed by the nature of the excited state and subsequent energy redistribution. The correlated growth kinetics observed here indicate that these products originate from the same excited-state intermediate rather than from sequential ground-state reactions. In addition, studies of related photochemical rearrangements have demonstrated that product formation may occur either directly from the excited state or via vibrationally hot intermediates. This behavior reflects competition on the excited-state potential energy surface. The absence of detectable ground-state intermediates in the present system supports a mechanism in which both products are formed before full relaxation. In addition to these product-related features, two transient spectral feature sets, a band at 1825 cm^−1^ and two bands at 1878 and 1845 cm^−1^, are observed. These features resemble those of 3-phenyl-1,4,2-dioxazol-5-one, suggesting the presence of excited-state intermediates. To quantitatively describe the spectral evolution and extract kinetic information, the entire TRIR dataset was analyzed by global fitting.

In the global fitting analysis, the time-dependent spectra were expressed as a linear combination of time-independent basis spectra with time-dependent amplitudes. Six basis spectra were required to reproduce the experimental data: ground-state 3-phenyl-1,4,2-dioxazol-5-one bleach, its S_1_ and T_1_ excited states, benzoyl nitrene, phenyl isocyanate, and CO_2_ (Figure 3a). The basis spectra for the reactant, phenyl isocyanate, and CO_2_ were obtained directly from equilibrium FTIR measurements. The benzoyl nitrene spectrum was extracted by fitting the TRIR data in the 1800–1700 cm^−1^ region, where its characteristic bands are isolated. The spectra corresponding to the S_1_ and T_1_ states were constructed from calculated vibrational stick spectra obtained from TD-DFT calculations at the ωB97X-D/aug-cc-pVTZ level. These spectra were scaled by factors of 0.96–0.97 and subsequently refined during the global fitting procedure. Specifically, the peak positions, bandwidths, and relative intensities were optimized to reproduce the experimental spectra. Initially, the integrated intensities of individual bands were constrained to follow the calculated values; in the final fitting, these constraints were relaxed to achieve optimal agreement with the experimental data.

**Figure 4:**
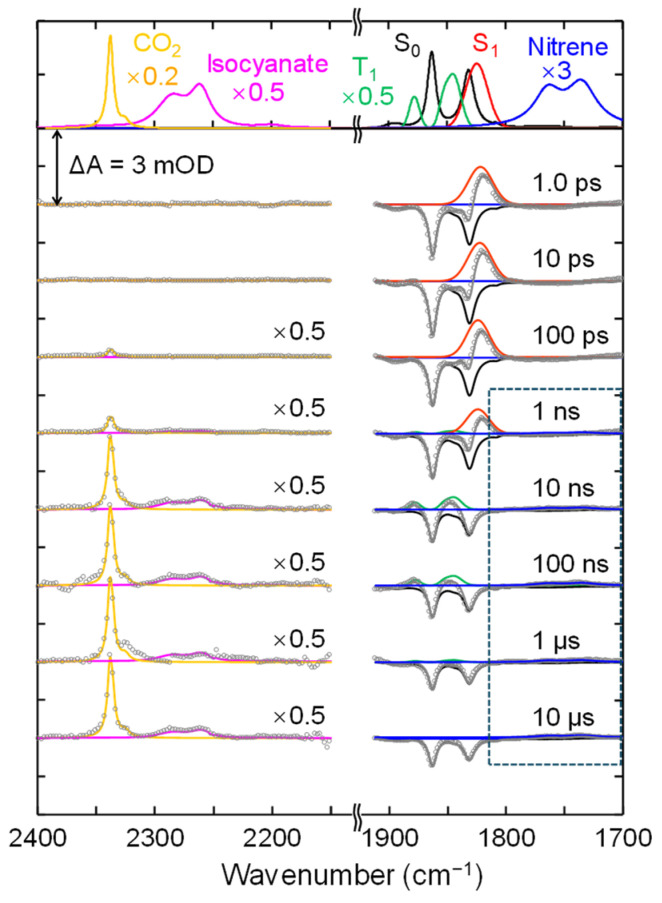
Representative TRIR spectra at selected delay times (open circles) and corresponding fits (gray solid lines) obtained from a linear combination of the six basis spectra shown above and in Figure 3a. For clarity, the spectra in the 2400–2200 cm^−1^ region are scaled by a factor of 0.5. The 1820–1700 cm^−1^ region, which contains weak but diagnostic features, is highlighted by a dashed rectangle and expanded in Figure 2c.

**Figure 5:**
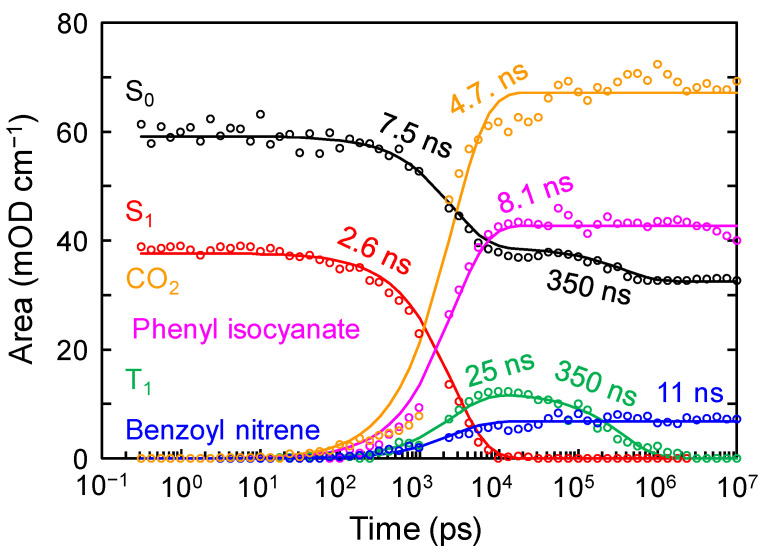
Time-dependent amplitudes of the six basis spectra obtained from global fitting: ground-state reactant (S_0_, black), S_1_ (red), T_1_ (green), benzoyl nitrene (blue), phenyl isocyanate (pink), and CO_2_ (orange). The reactant signal represents ground-state depletion, whereas all other signals correspond to transient absorptions. Solid lines represent the exponential fits used to extract the time constants and are color-coded.

The global fitting reproduced the experimental TRIR spectra across the entire time and frequency domains (Figure 2b and Figure 4), validating the choice of basis spectra. The time-dependent amplitudes of the basis spectra (Figure 5) provide direct access to the kinetics of each species. The band at 1825 cm^−1^, assigned to the S_1_ state, decayed with a time constant of 2.6 ± 1 ns. This decay was accompanied by the recovery of the bleach (τ = 7.5 ± 2 ns), the growth of CO_2_ (τ = 4.7 ± 1 ns), that is related to the growth of phenyl isocyanate (τ = 8.1 ± 2 ns) and benzoyl nitrene (τ = 11 ± 3 ns), and the growth of the T_1_ state (τ = 25 ± 5 ns), indicating that IC, photodecarboxylation, and ISC proceed from the S_1_ state. Two bands at 1878 and 1845 cm^−1^, assigned to the T_1_ state, decayed with a time constant of 350 ± 30 ns. The recovery of the ground-state bleach followed the same time constant, indicating that the T_1_ state relaxes to the ground state. Notably, the evolution of the CO_2_-related band is decoupled from the T_1_ dynamics, confirming that decarboxylation occurs from the S_1_ state before ISC.

**Figure 6:**
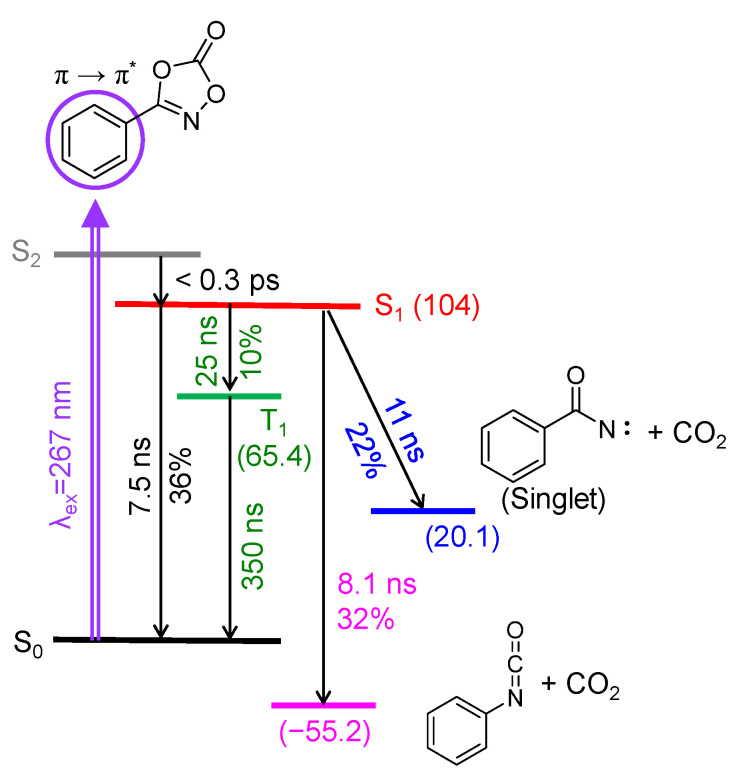
Proposed kinetic scheme for 3-phenyl-1,4,2-dioxazol-5-one in CHCl_3_ at 293 K following excitation at 267 nm, constructed from the amplitude kinetics of the basis spectra. The time constants were obtained from global analysis. The S_1_ state serves as the central branching point for internal conversion, intersystem crossing, and decarboxylation leading to product formation. The relative Gibbs free energies of each state are indicated in parentheses in kcal/mol.

Based on these observations, a kinetic model is proposed (Figure 6). According to this scheme, photoexcitation at 267 nm populates the S_2_ state, which undergoes ultrafast IC (<0.3 ps) to the S_1_ state. The S_1_ state serves as a branching point for three competing pathways: (i) IC to the ground state (τ = 7.5 ± 2 ns), (ii) ISC to the T_1_ state (τ = 25 ± 5 ns), and (iii) decarboxylation to yield either phenyl isocyanate (τ = 8.1 ± 2 ns) or benzoyl nitrene (τ = 11 ± 3 ns), accompanied by CO_2_ release (τ = 4.7 ± 1 ns). The T_1_ state subsequently relaxes to the ground state with a time constant of 350 ± 30 ns. Although phenyl isocyanate and benzoyl nitrene remain unreactive throughout the experimental time window of up to 10 μs, 3-phenyl-1,4,2-dioxazol-5-one in the T_1_ state decays with a time constant of 350 ± 30 ns, regenerating 3-phenyl-1,4,2-dioxazol-5-one in the S_0_ state. Importantly, both phenyl isocyanate and benzoyl nitrene are formed from the S_1_ state, establishing it as the key branching point governing product formation. The benzoyl nitrene produced under these conditions remains stable on the microsecond timescale, consistent with the reported lifetime of singlet benzoyl nitrene in CH_2_Cl_2_, approximately 6.25 μs [5,9,12,13]. Phenyl isocyanate and benzoyl nitrene are formed from the S_1_ state with distinct time constants, indicating competing pathways on the excited-state potential energy surface. This mechanistic picture is consistent with previous ultrafast studies of acyl azides and extends the excited-state branching model to dioxazolones.

## 3. Materials and Methods

### 3.1. Sample Preparation

3-phenyl-1,4,2-dioxazol-5-one was synthesized following literature procedures [17]. CHCl_3_ and phenyl isocyanate were purchased from Sigma-Aldrich (St. Louis, MO, USA) and used without further purification. A 20 mM solution of 3-phenyl-1,4,2-dioxazol-5-one was prepared in CHCl_3_ that had been thoroughly degassed by bubbling with N_2_ gas to remove dissolved O_2_. All sample preparation was carried out under a nitrogen atmosphere to minimize exposure to moisture and oxygen. The samples were loaded into a gas-tight flowing sample cell with a 100 μm path length and two 2-mm-thick CaF_2_ windows. The concentration and path length were adjusted to provide a sufficient transient absorption signal without introducing excessive optical density, which could cause pump-induced nonlinear absorption effects [26]. FTIR and UV–vis spectra were recorded before and after each measurement to confirm the chemical integrity of the samples. The temperature was maintained at 293 ± 1 K by circulating coolant through an aluminum block attached to the sample cell.

### 3.2. TRIR Spectroscopy

Mid-infrared transient absorption spectra were measured using a femtosecond pump–probe setup with temporal delays spanning from subpicoseconds to several microseconds. Details of the TRIR spectrometer used in this study have been described elsewhere [27,28,29]. Briefly, 120 fs laser pulses at 800 nm with a repetition rate of 2 kHz were generated using a commercial Ti:sapphire regenerative amplifier (Spitfire Ace, Spectra Physics (Milpitas, CA, USA)). The 800 nm output was split and directed into a home-built optical parametric amplifier (OPA) and a third-harmonic generator (THG). The OPA generated signal and idler beams, which were used to produce mid-infrared probe pulses, approximately 120 fs in duration, with a bandwidth of approximately 160 cm^−1^, and a pulse energy of approximately 1 μJ via difference-frequency mixing in a 1-mm-thick type-I AgGaS_2_ crystal. The THG produced 267 nm pump pulses, approximately 150 fs in duration, with a pulse energy of approximately 10 μJ. For time delays up to 1 ns, a mechanical delay stage was used to control the temporal overlap between the UV pump and mid-IR probe pulses. For longer delays, the femtosecond UV pump was replaced with a nanosecond tunable laser (NT240, EKSPLA, Vilnius, Lithuania; pulse width, approximately 2.4 ns), synchronized with the femtosecond mid-IR probe using a digital delay generator (DG535, Stanford Research Systems (Sunnyvale, CA, USA)). The pump pulse energy was set to 1 μJ for the femtosecond pump or 3 μJ for the nanosecond pump, with a spot size of approximately 200 μm, to ensure low excitation fluence and linear response conditions. The mid-IR probe pulse energy, approximately 10 nJ, was kept sufficiently low to avoid perturbing the sample. To obtain isotropic signals unaffected by rotational diffusion, the pump polarization was set at the magic angle, 54.7°, relative to the probe polarization. The mid-IR probe was tuned to selected spectral regions, 2250, 1850, 1780, and 1750 cm^−1^, to extend coverage beyond the intrinsic bandwidth of the pulse, approximately 160 cm^−1^. After passing through the sample, the probe beam was dispersed by a 320 mm monochromator (HR320, Horiba, Miami, FL, USA) equipped with a 150 lines/mm grating and detected using a 1 × 128 pixel HgCdTe array detector (MCT-8-128, Infrared Associates, Stuart, FL, USA). The detector output was amplified and digitized with 16-bit analog-to-digital converters, yielding a spectral resolution of approximately 1.5 cm^−1^ per pixel. For signal acquisition, a 1 kHz optical chopper (MC1000A, Thorlabs, Newton, NJ, USA) modulated the 2 kHz pump pulses, enabling quasi-simultaneous differential detection of the pumped and unpumped states. In the nanosecond experiments, the pump operated at 1 kHz without chopping. The instrument response function was determined from the cross-correlation of the pump and probe pulses using a Si wafer, yielding temporal resolutions of approximately 0.2 ps for femtosecond excitation and approximately 2.5 ns for nanosecond excitation.

### 3.3. Computational Details

The structures of the ground state, excited states, and photoproducts were optimized using DFT and TD-DFT with the ωB97X-D functional and the aug-cc-pVTZ basis set [30], as implemented in Gaussian 16 [31]. The solvent effects of CHCl_3_ were included using the polarizable continuum model [32]. Anharmonic vibrational frequencies were calculated for the optimized structures and scaled to match the experimental values [33]. This level of theory was selected to provide reliable predictions of vibrational coupling and mode mixing relevant to the observed resonance features.

## 4. Conclusions

The ultrafast photochemistry of 3-phenyl-1,4,2-dioxazol-5-one in CHCl_3_ was elucidated using femtosecond TRIR spectroscopy in combination with electronic structure calculations and global kinetic analysis. Following excitation at 267 nm, rapid IC (<0.3 ps) populates the S_1_ state, which serves as the central branching point governing both photophysical relaxation and photochemical transformation. Global analysis of the TRIR dataset, based on a linear combination of six basis spectra, enabled direct identification and kinetic characterization of all major species, including the reactant, its S_1_ and T_1_ states, CO_2_, phenyl isocyanate, and benzoyl nitrene. The results showed that decarboxylation occurred from the S_1_ state on the nanosecond timescale, producing CO_2_ together with phenyl isocyanate and singlet benzoyl nitrene, whereas competing pathways included IC to the ground state and ISC to the T_1_ state. The T_1_ state relaxed to the ground state without contributing to product formation. A key finding of this work is the unambiguous spectroscopic identification of singlet benzoyl nitrene through its characteristic C=N stretching bands in the 1800–1700 cm^−1^ region, supported by vibrational calculations. The absence of signatures associated with triplet nitrene indicates that nitrene formation proceeds predominantly on the singlet manifold under the present conditions. Furthermore, the benzoyl nitrene intermediate was stable on the microsecond timescale in solution. These results establish that both isocyanate and nitrene products originate directly from the S_1_ state, demonstrating that dioxazolone photochemistry is governed by excited-state dynamics rather than ground-state processes. This behavior closely parallels that observed in acyl azides and related systems, while extending the excited-state branching framework to dioxazolones. Overall, this work provides a comprehensive, time-resolved mechanistic picture linking electronic excitation, vibrational signatures, and product formation. The findings highlight the critical role of energy flow and excited-state evolution in determining reaction pathways and demonstrate the power of ultrafast vibrational spectroscopy in resolving competing photochemical processes in solution. These insights offer a general framework for understanding and controlling nitrene generation from carbonyl-containing precursors.

## Data Availability

The data supporting the findings of this study are included in this paper and Supporting Information. Should any raw data files be required in another format, they are available from the corresponding author upon reasonable request. Source data are provided with this paper and Supporting Information.

## Supplementary Materials

The following supporting information can be downloaded at https://www.mdpi.com/article/10.3390/ijms27125563/s1.

## Abbreviations

The following abbreviations are used in this manuscript:

TRIR	Time-resolved infrared
TD-DFT	Time-dependent density functional theory
IC	Internal conversion
ISC	Intersystem crossing
FTIR	Fourier transform infrared
OPA	Optical parametric amplifier
THG	Third-harmonic generator
DFT	Density functional theory
OD	Optical density
UV–vis	Ultraviolet–visible

## References

[1] Du Y., Yu X., Tang J.-J., Li Y., Fan J., Li F., Bao M. (2023). Visible-light-promoted Mn-catalyzed C(sp^3^)–H amidation with dioxazolones. J. Org. Chem..

[2] Tang J.-J., Yu X., Yamamoto Y., Bao M. (2021). Visible-light-promoted iron-catalyzed *N*-arylation of dioxazolones with arylboronic acids. ACS Catal..

[3] Park J., Krishnapriya A.U., Park Y., Kim M., Reidl T.W., Kuniyil R., Son J. (2023). Copper(I)-catalyzed decarboxylative *N*-phosphorylation: Modular preparation of *N*-acyl iminophosphoranes using dioxazolones and phosphines. Adv. Synth. Catal..

[4] van Vliet K.M., de Bruin B. (2020). Dioxazolones: Stable substrates for the catalytic transfer of acyl nitrenes. ACS Catal..

[5] Kubicki J., Zhang Y., Wang J., Luk H.L., Peng H.-L., Vyas S., Platz M.S. (2009). Direct observation of acyl azide excited states and their decay processes by ultrafast time resolved infrared spectroscopy. J. Am. Chem. Soc..

[6] West M.J., Fyfe J.W.B., Vantourout J.C., Watson A.J.B. (2019). Mechanistic development and recent applications of the chan–lam amination. Chem. Rev..

[7] Dequirez G., Pons V., Dauban P. (2012). Nitrene chemistry in organic synthesis: Still in its infancy?. Angew. Chem. Int. Ed..

[8] Dam D., Lagerweij N.R., Janmaat K.M., Kok K., Bouwman E., Codée J.D.C. (2024). Organic dye-sensitized nitrene generation: Intermolecular aziridination of unactivated alkenes. J. Org. Chem..

[9] Kubicki J., Zhang Y., Vyas S., Burdzinski G., Luk H.L., Wang J., Xue J., Peng H.-L., Pritchina E.A., Sliwa M. (2011). Photochemistry of 2-naphthoyl azide. An ultrafast time-resolved UV–Vis and IR spectroscopic and computational study. J. Am. Chem. Soc..

[10] Gritsan N.P. (2007). Study of photochemical transformations of organic azides by matrix isolation techniques and quantum chemistry. Russ. Chem. Rev..

[11] Gritsan N.P., Platz M.S. (2006). Kinetics, spectroscopy, and computational chemistry of arylnitrenes. Chem. Rev..

[12] Pritchina E.A., Gritsan N.P., Maltsev A., Bally T., Autrey T., Liu Y., Wang Y., Toscano J.P. (2003). Matrix isolation, time-resolved IR, and computational study of the photochemistry of benzoyl azide. Phys. Chem. Chem. Phys..

[13] Sigman M.E., Autrey T., Schuster G.B. (1988). Aroylnitrenes with singlet ground states: Photochemistry of acetyl-substituted aroyl and aryloxycarbonyl azides. J. Am. Chem. Soc..

[14] Autrey T., Schuster G.B. (1987). Are aroylnitrenes ground-state singlets? Photochemistry of.Beta.-naphthoyl azide. J. Am. Chem. Soc..

[15] Liu J., Mandel S., Hadad C.M., Platz M.S. (2004). A comparison of acetyl- and methoxycarbonylnitrenes by computational methods and a laser flash photolysis study of benzoylnitrene. J. Org. Chem..

[16] Sun H., Zhu B., Wu Z., Zeng X., Beckers H., Jenks W.S. (2015). Thermally persistent carbonyl nitrene: FC(O)N. J. Org. Chem..

[17] Adegboyega A.K., Son J. (2022). Reaction of dioxazolones with boronic acids: Copper-mediated synthesis of *N*-aryl amides via *N*-acyl nitrenes. Org. Lett..

[18] Gong S., Wang S.-H., Wang Z.-L., He W.-M. (2025). Recent advances in photocatalytic transformations of dioxazolones. Adv. Synth. Catal..

[19] Burdzinski G.T., Wang J., Gustafson T.L., Platz M.S. (2008). Study of concerted and sequential photochemical wolff rearrangement by femtosecond UV−Vis and IR spectroscopy. J. Am. Chem. Soc..

[20] Yoon H., Park S., Lim M. (2023). Dynamics of irreversible NO release from photoexcited molsidomine. J. Phys. Chem. Lett..

[21] Runge E., Gross E.K.U. (1984). Density-functional theory for time-dependent systems. Phys. Rev. Lett..

[22] Chai J.-D., Head-Gordon M. (2008). Long-range corrected hybrid density functionals with damped atom–atom dispersion corrections. Phys. Chem. Chem. Phys..

[23] Dunning T.H. (1989). Gaussian basis sets for use in correlated molecular calculations. I. The atoms boron through neon and hydrogen. J. Chem. Phys..

[24] Chantry G.W., Nicol E.A., Harrison D.J., Bouchy A., Roussy G. (1974). Vibrational spectrum, assignment and molecular symmetry of phenyl isocyanate in the liquid phase. Spectrochim. Acta A Mol. Spectrosc..

[25] Doddamani S.B., Ramoji A., Yenagi J., Tonannavar J. (2007). The vibrational spectra, assignments and ab initio/DFT analysis for 3-chloro, 4-chloro and 5-chloro-2-methylphenyl isocyanates. Spectrochim. Acta A Mol. Biomol. Spectrosc..

[26] Berera R., van Grondelle R., Kennis J.T.M. (2009). Ultrafast transient absorption spectroscopy: Principles and application to photosynthetic systems. Photosynth. Res..

[27] Park S., Yoon H., Shin J., Lim M. (2025). Determination of the rotational isomerization rate along carbon–carbon single bonds in solution. Phys. Chem. Chem. Phys..

[28] Yoon H., Park S., Kim S.Y., Hong D., Park J.W., Lim M. (2024). Dynamics of NO release and linkage isomer formation from S-nitroso-mercaptoethanol in aqueous solutions: Insights from femtosecond infrared spectroscopy. J. Phys. Chem. Lett..

[29] Yoon H., Shin J., Park S., Lim M. (2025). Ultrafast photochemical dynamics of dinitrosyl iron complexes investigated by femtosecond time-resolved infrared spectroscopy. Int. J. Mol. Sci..

[30] Kendall R.A., Dunning T.H., Harrison R.J. (1992). Electron affinities of the first-row atoms revisited. Systematic basis sets and wave functions. J. Chem. Phys..

[31] Frisch M.J., Trucks G.W., Schlegel H.B., Scuseria G.E., Robb M.A., Cheeseman J.R., Scalmani G., Barone V., Petersson G.A., Nakatsuji H. (2016). Gaussian 16 Rev. C.01.

[32] Scalmani G., Frisch M.J. (2010). Continuous surface charge polarizable continuum models of solvation. I. General formalism. J. Chem. Phys..

[33] Barone V. (2004). Anharmonic vibrational properties by a fully automated second-order perturbative approach. J. Chem. Phys..

