# Supplementary material for: Ultrafast Photochemical Reaction Dynamics of 3-Phenyl-1,4,2-dioxazol-5-one Revealed by Femtosecond Time-Resolved Infrared Spectroscopy
Source: Int J Mol Sci. 2026 Jun 19;27(12):5563. doi: 10.3390/ijms27125563 (PMC13299421; doi:10.3390/ijms27125563)
Supplement: Supplementary file 1 [file ijms-27-05563-s001.zip › ijms-4374165-supplementary.pdf]

Supporting Information

Table S1. TD-DFT vertical excitation energies, wavelengths, oscillator strengths, and orbital contributions for the five lowest singlet excited states of 3-phenyl-1,4,2-dioxazol-5-one calculated using the ωB97X-D functional and the aug-cc-pVTZ basis set. The corresponding molecular orbitals are shown in Figure S1.

State	Energy (eV)	Wavelength (nm)	Oscillator strength	Transition: coefficient
S_1	5.0871	243.72	0.0304	HOMO−1 → LUMO: 0.59683<br>HOMO → LUMO+2: 0.31600<br>HOMO → LUMO: -0.14440<br>HOMO−2 → LUMO+2: 0.10916
S_2	5.2154	237.73	0.4955	HOMO → LUMO: 0.67303<br>HOMO−1 → LUMO: 0.11807<br>HOMO−1 → LUMO+2: 0.10235
S_3	6.4209	193.09	0.3178	HOMO → LUMO+2: 0.58020<br>HOMO−1 → LUMO: 0.34418<br>HOMO−2 → LUMO+2: 0.10190
S_4	6.5281	189.93	0.3868	HOMO−1 → LUMO+2: 0.56906<br>HOMO−2 → LUMO: 0.35022<br>HOMO → LUMO+2: 0.11797<br>HOMO → LUMO+14: 0.11797
S_5	6.7188	184.53	0.0040	HOMO−3 → LUMO: 0.56683<br>HOMO−4 → LUMO: 0.27377<br>HOMO−6 → LUMO: -0.20342<br>HOMO−3 → LUMO+14: -0.12916

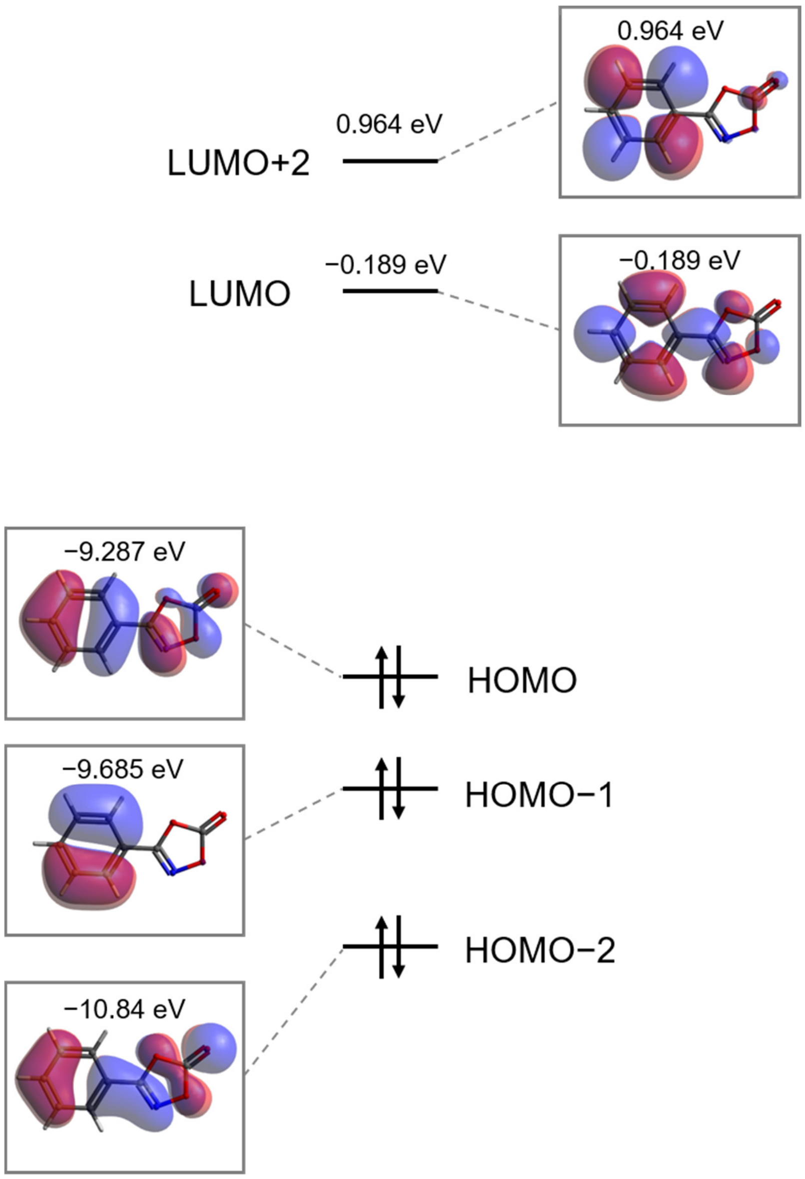

Figure S1. TD-DFT-calculated molecular orbitals involved in the electronic transitions of 3-phenyl-1,4,2-dioxazol-5-one at the ωB97X-D/aug-cc-pVTZ level.

Table S2. Harmonic IR frequencies of S_0, S_1, and T_1 3-phenyl-1,4,2-dioxazol-5-one calculated at the ωB97X-D/aug-cc-pVTZ level. Vibrational frequencies were scaled by factors of 0.9812, 0.9619, and 0.9760 for the S_0, S_1, and T_1 states, respectively. Because only a single band was observed in the experimental spectrum of S_1 3-phenyl-1,4,2-dioxazol-5-one, harmonic frequencies were sufficient to reproduce the observed spectral features. Frequencies used for assignment of the experimental spectrum are highlighted in bold.

	S_0 geometry		S_1 geometry		T_1 geometry	
Vibration mode	Frequency (cm^{-1})	Intensity (km/mol)	Frequency (cm^{-1})	Intensity (km/mol)	Frequency (cm^{-1})	Intensity (km/mol)
ν_1	35.39	0.0163	47.73	0.2248	54.93	0.3162
ν_2	95.22	0.8940	110.07	0.0007	115.54	0.0594
ν_3	130.17	0.7053	114.78	0.4120	120.62	0.5176
ν_4	208.78	4.0509	141.09	0.3310	140.84	0.1872
ν_5	320.82	0.5547	257.29	6.6087	244.08	4.0271
ν_6	323.70	1.5976	310.45	13.4902	315.36	0.3996
ν_7	395.46	0.7512	330.26	0.3020	357.81	1.1247
ν_8	403.12	0.0049	386.48	0.6202	388.10	19.1045
ν_9	487.60	3.7344	391.18	0.0996	401.73	0.1254
ν_{10}	580.36	10.8999	490.71	0.1024	494.95	8.5166
ν_{11}	622.46	0.2239	542.95	43.5163	558.69	10.5172
ν_{12}	683.04	16.6850	563.30	8.8920	594.33	4.9475
ν_{13}	697.65	15.4226	599.79	104.2975	633.64	68.5152
ν_{14}	699.86	63.7918	629.91	53.4610	640.58	89.1296
ν_{15}	768.29	1.4422	730.28	53.3017	741.93	53.0855
ν_{16}	771.61	71.4745	732.28	23.0912	744.65	47.6550
ν_{17}	799.19	3.4436	739.31	5.0615	750.41	31.2024
ν_{18}	863.80	0.0005	746.23	10.9328	776.66	0.0000
ν_{19}	926.78	10.1690	841.77	3.3096	788.14	132.0687
ν_{20}	951.00	9.4131	862.30	6.3644	853.03	7.8685
ν_{21}	958.19	2.4654	912.56	29.9762	859.17	56.8886
ν_{22}	1004.38	0.0089	946.62	2.4482	955.23	119.1892
ν_{23}	1009.09	39.5845	955.92	26.3003	979.86	0.2101
ν_{24}	1011.51	153.4637	962.90	0.1597	985.68	0.3968
ν_{25}	1026.43	0.0007	969.84	0.1074	986.94	1.4533
ν_{26}	1040.21	50.4093	977.06	838.8840	1007.90	32.8683
ν_{27}	1094.21	119.1243	1042.08	154.7621	1049.71	211.2801
ν_{28}	1100.99	17.8573	1070.39	76.4551	1084.13	37.6026
ν_{29}	1169.51	3.9030	1100.37	97.8282	1122.85	38.5768
ν_{30}	1190.96	62.9510	1116.69	238.6150	1150.56	1019.4431
ν_{31}	1196.38	53.1341	1163.75	9.4167	1180.59	139.0391
ν_{32}	1310.56	18.7768	1215.02	139.3783	1202.16	113.4200
ν_{33}	1342.16	6.5740	1275.90	488.9229	1308.08	28.9710
ν_{34}	1383.35	297.1896	1332.93	4.9545	1314.85	15.8621
ν_{35}	1471.06	38.4597	1369.87	26.2116	1404.20	16.6034
ν_{36}	1522.30	14.3635	1405.44	54.7999	1434.02	49.1477

ν_{37}	1618.18	32.6757	1482.64	23.0280	1523.56	15.7525
ν_{38}	1647.19	8.1860	1522.36	9.9981	1545.40	30.6017
ν_{39}	1669.71	177.0359	1646.24	8.5610	1664.38	206.3375
ν_{40}	1875.03	1183.2340	**1824.67**	**1837.5226**	1881.48	1550.9771
ν_{41}	3141.89	0.1392	3083.50	0.8433	3124.49	1.1594
ν_{42}	3151.68	4.9938	3089.96	10.8105	3128.90	10.5388
ν_{43}	3158.01	8.0694	3098.95	8.8232	3145.21	11.2997
ν_{44}	3165.96	8.3572	3108.66	4.8017	3150.98	9.5013
ν_{45}	3170.41	7.5872	3111.91	9.6297	3155.12	14.6423

Table S3. Anharmonic vibrational frequencies of S_0 and T_1 3-phenyl-1,4,2-dioxazol-5-one calculated at the ωB97X-D/aug-cc-pVTZ level within the spectral region investigated by TRIR spectroscopy. Vibrational frequencies were scaled by factors of 0.9812 and 0.9760 for the S_0 and T_1 states, respectively, to match the experimental spectra. IR bands with calculated intensities greater than 25 km/mol (S_0) and 140 km/mol (T_1) are highlighted in bold and were used for assignment of the experimental spectra.

S_0 geometry			T_1 geometry		
Vibration mode	Frequency (cm^{-1})	Intensity (km/mol)	Vibration mode	Frequency (cm^{-1})	Intensity (km/mol)
Over(ν_{19})	1787.13	18.1768	Comb(ν_{21} ν_{22})	1780.24	0.1968
Comb(ν_{5} ν_{36})	1788.43	1.5155	Comb(ν_{9} ν_{36})	1780.75	0.0002
Comb(ν_{12} ν_{29})	1789.80	0.4124	Comb(ν_{13} ν_{31})	1782.05	0.0002
Comb(ν_{17} ν_{26})	1798.26	0.0177	Comb(ν_{20} ν_{22})	1785.81	0.0003
Comb(ν_{7} ν_{35})	1802.04	2.3107	Comb(ν_{16} ν_{28})	1787.38	0.0881
**Comb(ν_{15} ν_{28})**	**1806.81**	**27.8396**	Comb(ν_{21} ν_{25})	1796.76	0.0888
Comb(ν_{12} ν_{30})	1807.37	0.7121	Comb(ν_{17} ν_{28})	1797.58	0.0002
**Comb(ν_{15} ν_{27})**	**1810.39**	**67.6499**	Comb(ν_{20} ν_{25})	1798.46	0.0061
Comb(ν_{17} ν_{25})	1812.69	0.1174	Comb(ν_{19} ν_{28})	1799.15	6.3761
Comb(ν_{4} ν_{38})	1812.77	0.0055	Comb(ν_{21} ν_{23})	1805.28	0.0002
Comb(ν_{16} ν_{27})	1814.97	0.0009	Comb(ν_{18} ν_{28})	1806.79	0.0194
Comb(ν_{18} ν_{22})	1815.31	4.5807	Comb(ν_{6} ν_{38})	1807.95	0.3152
Comb(ν_{9} ν_{34})	1823.00	0.0016	Comb(ν_{20} ν_{23})	1809.34	0.8115
Comb(ν_{8} ν_{35})	1825.86	0.0001	Comb(ν_{11} ν_{34})	1812.75	6.1867
Comb(ν_{18} ν_{24})	1828.52	0.0002	Comb(ν_{21} ν_{26})	1826.95	44.4248
Comb(ν_{13} ν_{30})	1829.50	0.0001	Comb(ν_{21} ν_{24})	1829.69	0.0004
Comb(ν_{18} ν_{23})	1831.19	0.0001	Comb(ν_{11} ν_{33})	1829.87	8.0992
**Comb(ν_{19} ν_{20})**	**1831.94**	**37.3029**	Comb(ν_{20} ν_{26})	1832.10	0.0012
**Comb(ν_{12} ν_{31})**	**1833.92**	**296.5747**	Comb(ν_{20} ν_{24})	1833.74	2.6083
Comb(ν_{14} ν_{30})	1836.98	0.0010	Comb(ν_{16} ν_{29})	1834.23	4.4428
Comb(ν_{17} ν_{28})	1838.78	0.0001	ν_{40}	**1839.82**	**509.7394**
Comb(ν_{4} ν_{39})	1841.88	0.0095	Comb(ν_{19} ν_{29})	1844.22	2.0405
Comb(ν_{17} ν_{27})	1842.19	0.0032	Comb(ν_{12} ν_{34})	1850.71	0.1831
**ν_{40}**	**1842.79**	**276.5810**	Comb(ν_{7} ν_{37})	1852.58	0.0013
Comb(ν_{19} ν_{21})	1843.95	0.0005	Comb(ν_{21} ν_{27})	1853.52	77.0297
Comb(ν_{10} ν_{33})	1849.77	0.0929	Comb(ν_{20} ν_{27})	1855.11	0.0017
Comb(ν_{10} ν_{32})	1850.00	0.0969	Comb(ν_{8} ν_{37})	1856.58	0.0984
Comb(ν_{7} ν_{36})	1855.02	0.0320	Comb(ν_{16} ν_{30})	**1859.42**	**201.8074**

Comb(ν_{18} ν_{25})	1858.18	0.3199	Comb(ν_{17} ν_{30})	1863.52	0.0007
Comb(ν_{14} ν_{31})	1863.13	0.0001	Comb(ν_{19} ν_{30})	1865.70	1.1211
Comb(ν_{1} ν_{40})	1865.08	0.7359	Comb(ν_{12} ν_{33})	1866.97	0.0440
**Over(ν_{20})**	**1869.77**	**81.0047**	Comb(ν_{16} ν_{32})	1868.34	13.0640
Comb(ν_{11} ν_{33})	1874.79	0.0016	Comb(ν_{15} ν_{30})	1870.68	0.0001
Comb(ν_{5} ν_{37})	1875.55	0.0568	Comb(ν_{18} ν_{30})	1874.35	0.0001
Comb(ν_{11} ν_{32})	1876.22	0.0773	Comb(ν_{5} ν_{39})	1876.18	0.0063
Comb(ν_{8} ν_{36})	1877.72	0.0001	Comb(ν_{9} ν_{37})	1876.92	0.0096
Comb(ν_{15} ν_{29})	1879.06	0.0137	Over(ν_{22})	**1877.45**	**142.7056**
Comb(ν_{6} ν_{37})	1882.14	0.0083	Comb(ν_{10} ν_{35})	1877.71	0.0002
Comb(ν_{19} ν_{34})	1883.17	9.4289	Comb(ν_{19} ν_{32})	1879.24	15.9572
Over(ν_{21})	1887.65	0.0523	Comb(ν_{10} ν_{36})	1883.83	0.0052
Comb(ν_{18} ν_{28})	1889.88	0.0142	Comb(ν_{16} ν_{31})	1884.33	34.9869
Comb(ν_{19} ν_{23})	1890.33	1.9897	Comb(ν_{15} ν_{32})	1884.86	0.0002
Comb(ν_{18} ν_{27})	1894.01	0.0012	Comb(ν_{7} ν_{38})	1888.10	0.0038
Comb(ν_{15} ν_{30})	1896.75	0.3778	Comb(ν_{18} ν_{32})	1889.26	0.0024
Comb(ν_{16} ν_{30})	1899.83	0.0003	Comb(ν_{14} ν_{34})	1889.36	0.1170
Comb(ν_{9} ν_{35})	1901.43	0.0004	Comb(ν_{8} ν_{38})	1891.60	0.1383
Comb(ν_{19} ν_{26})	1907.86	2.6551	Comb(ν_{17} ν_{31})	1892.59	0.0002
Comb(ν_{5} ν_{38})	1914.32	0.0128	Comb(ν_{22} ν_{25})	1892.94	0.5468
Comb(ν_{6} ν_{38})	1918.89	0.0159	Comb(ν_{21} ν_{28})	1894.82	0.7274
Comb(ν_{10} ν_{34})	1920.49	0.6199	Comb(ν_{19} ν_{31})	1900.33	1.6558
Comb(ν_{15} ν_{31})	1921.00	0.6810	Comb(ν_{13} ν_{34})	1900.33	0.0005
Comb(ν_{19} ν_{25})	1921.66	0.0003	Comb(ν_{20} ν_{28})	1900.92	0.0001
Comb(ν_{20} ν_{24})	1922.27	12.5982	Comb(ν_{22} ν_{23})	1904.83	0.0008
Comb(ν_{16} ν_{31})	1924.12	0.0003	Comb(ν_{14} ν_{33})	1906.17	0.4520
Comb(ν_{21} ν_{22})	1925.50	1.6130	Over(ν_{25})	1906.61	0.0941
Comb(ν_{20} ν_{23})	1927.05	1.5128	Comb(ν_{6} ν_{39})	1909.03	0.9742
Comb(ν_{3} ν_{40})	1932.64	0.2337	Comb(ν_{9} ν_{38})	1911.92	0.0038
Comb(ν_{2} ν_{40})	1933.48	0.2380	Comb(ν_{13} ν_{33})	1915.49	0.0007
Comb(ν_{21} ν_{24})	1934.46	0.0034	Comb(ν_{11} ν_{35})	1915.82	0.4196
Comb(ν_{21} ν_{23})	1937.11	0.0064	Comb(ν_{23} ν_{25})	1916.31	0.0051
Comb(ν_{12} ν_{33})	1938.63	0.0018	Comb(ν_{1} ν_{40})	1916.80	0.6645
Comb(ν_{12} ν_{32})	1939.50	0.0211	Over(ν_{23})	1920.18	0.8248
Comb(ν_{5} ν_{39})	1943.09	0.4058	Comb(ν_{22} ν_{26})	1922.65	6.3457
Comb(ν_{20} ν_{26})	1944.44	0.0102	Comb(ν_{22} ν_{24})	1925.44	0.0065
Comb(ν_{7} ν_{37})	1945.18	0.0407	Comb(ν_{11} ν_{36})	1925.58	0.0050
Comb(ν_{6} ν_{39})	1947.43	0.0140	Comb(ν_{3} ν_{40})	1928.98	0.1421
Comb(ν_{11} ν_{34})	1948.16	0.0147	Comb(ν_{25} ν_{26})	1938.77	0.2420
Comb(ν_{19} ν_{28})	1948.28	1.4514	Comb(ν_{24} ν_{25})	1940.94	0.0291
Comb(ν_{19} ν_{27})	1951.78	13.0062	Comb(ν_{21} ν_{29})	1941.88	0.5060

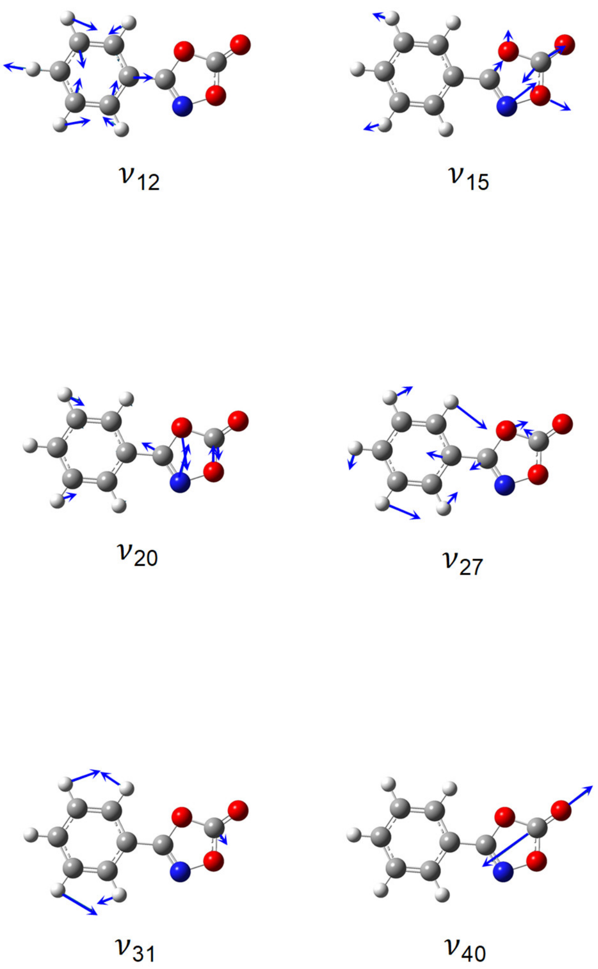

Figure S2. Selected vibration modes of S_0 3-phenyl-1,4,2-dioxazol-5-one listed in Table S3. These modes were used to assign the experimental spectrum of the S_0 state.

Table S4. Harmonic IR frequencies of singlet and triplet benzoyl nitrene, and phenyl isocyanate, calculated at the ωB97X-D/aug-cc-pVTZ level. Vibrational frequencies were scaled by factors of 0.9574 and 0.9896 for the benzoyl nitrene and phenyl isocyanate, respectively.

	Benzoyl nitrene (singlet)		Benzoyl nitrene (triplet)		Phenyl isocyanate	
Vibration mode	Frequency (cm^{-1})	Intensity (km/mol)	Frequency (cm^{-1})	Intensity (km/mol)	Frequency (cm^{-1})	Intensity (km/mol)
ν_1	76.94	0.0244	51.66	0.1575	52.07	0.0146
ν_2	137.02	1.9473	148.64	0.9668	84.15	4.1483
ν_3	150.22	5.2942	191.34	3.3495	240.33	0.0146
ν_4	354.87	1.7573	343.75	5.6474	386.04	19.4287
ν_5	393.13	0.0409	392.19	0.1498	418.36	0.0035
ν_6	399.04	0.5024	415.60	0.1025	466.84	2.3127
ν_7	490.55	0.0150	462.88	1.4329	507.12	8.5972
ν_8	555.07	23.1197	553.42	51.9973	590.45	29.7410
ν_9	605.35	48.3385	605.92	0.2544	627.14	2.5101
ν_{10}	605.80	0.4685	635.71	53.5412	650.01	53.7874
ν_{11}	679.78	59.8711	675.67	83.4699	705.27	41.1288
ν_{12}	748.91	1.3894	733.90	2.5078	774.00	18.6904
ν_{13}	768.21	33.2979	772.87	19.6382	776.59	80.3190
ν_{14}	842.80	0.0117	844.20	0.0158	853.82	0.0772
ν_{15}	937.64	2.1630	939.61	1.3251	935.22	6.7280
ν_{16}	979.03	0.0221	980.36	0.0320	996.16	0.0568
ν_{17}	984.69	0.7921	984.07	1.8224	1017.16	0.1252
ν_{18}	1002.78	0.0059	999.99	0.0020	1019.93	0.1229
ν_{19}	1011.99	5.9069	1010.65	14.9888	1047.54	12.0757
ν_{20}	1065.24	22.6156	1042.62	27.0581	1101.40	18.0541
ν_{21}	1131.83	31.0948	1073.87	4.6979	1151.30	74.9928
ν_{22}	1141.16	2.2388	1140.50	2.9572	1171.92	0.0222
ν_{23}	1158.47	7.8096	1154.60	37.3267	1194.02	0.1541
ν_{24}	1207.65	59.1606	1205.71	205.7551	1304.68	10.1676
ν_{25}	1289.19	2.9140	1286.30	23.9617	1348.34	0.1903
ν_{26}	1309.22	11.7324	1305.53	12.3824	1481.98	7.1778
ν_{27}	1438.17	25.5207	1434.76	26.3289	1488.28	8.1514
ν_{28}	1475.71	9.1599	1473.54	2.6523	1564.84	83.0101
ν_{29}	1586.40	0.8083	**1549.86**	**307.7505**	1637.08	15.3138
ν_{30}	1603.43	23.4242	1586.52	9.6945	1656.01	108.9017
ν_{31}	1777.35	440.0976	**1599.25**	**65.7776**	2324.93	2768.0785
ν_{32}	3066.37	0.0732	3062.81	0.0332	3163.75	2.0871
ν_{33}	3073.06	0.7443	3069.29	2.6436	3170.52	2.9869
ν_{34}	3076.69	4.9149	3076.67	7.5132	3178.93	18.2183
ν_{35}	3086.94	11.1630	3085.47	11.0741	3186.39	17.9874
ν_{36}	3092.47	8.6153	3090.69	11.7383	3193.11	5.4321

Table S5. Anharmonic vibrational frequencies of singlet benzoyl nitrene and phenyl isocyanate within the spectral region investigated by TRIR spectroscopy, calculated at the ωB97X-D/aug-cc-pVTZ level. Vibrational frequencies were scaled by factors of 0.9574 and 0.9896 for singlet benzoyl nitrene and phenyl isocyanate, respectively. IR bands with calculated intensities greater than 80 km/mol (singlet benzoyl nitrene) and 40 km/mol (phenyl isocyanate) are highlighted in bold and used for assignment of the experimental spectra.

Singlet benzoyl nitrene			Phenyl isocyanate		
Vibration mode	Frequency (cm^{-1})	Intensity (km/mol)	Vibration mode	Frequency (cm^{-1})	Intensity (km/mol)
Comb(ν_7 ν_{24})	1663.75	15.5246	Comb(ν_8 ν_{30})	2202.61	0.0005
Comb(ν_{11} ν_{16})	1665.01	0.0952	Comb(ν_{19} ν_{23})	2203.81	0.0467
Comb(ν_8 ν_{22})	1675.59	0.0043	Comb(ν_{15} ν_{24})	2206.63	0.0001
Over(ν_{14})	1678.67	0.0645	Comb(ν_{20} ν_{21})	2211.95	0.5068
Comb(ν_{11} ν_{19})	1681.62	0.0006	Comb(ν_9 ν_{29})	2223.39	0.0798
Comb(ν_5 ν_{26})	1681.91	0.0043	Comb(ν_{12} ν_{26})	2224.25	10.1420
Comb(ν_8 ν_{23})	1686.85	0.0355	**Comb(ν_{12} ν_{27})**	**2226.33**	**42.4360**
Comb(ν_2 ν_{29})	1693.10	0.0005	Comb(ν_{13} ν_{26})	2226.82	0.0013
Comb(ν_{13} ν_{15})	1699.58	1.1182	Comb(ν_{13} ν_{27})	2230.54	0.0041
Comb(ν_{12} ν_{17})	1706.70	0.0483	Comb(ν_9 ν_{30})	2234.69	0.9629
Comb(ν_2 ν_{30})	1707.48	0.0266	Comb(ν_{10} ν_{29})	2245.44	1.8654
Comb(ν_{11} ν_{18})	1710.61	0.0434	Over(ν_{21})	2246.96	9.6092
Comb(ν_9 ν_{21})	1713.07	0.0088	Comb(ν_{11} ν_{28})	2249.93	0.0038
Comb(ν_{10} ν_{21})	1713.86	0.8932	Comb(ν_{20} ν_{22})	2253.37	0.0274
Comb(ν_3 ν_{29})	1716.35	1.3645	Comb(ν_{15} ν_{25})	2253.60	0.0004
Comb(ν_8 ν_{24})	1726.40	2.5758	**Comb(ν_{10} ν_{30})**	**2256.52**	**86.7327**
Comb(ν_9 ν_{22})	1730.73	0.0390	Comb(ν_{20} ν_{23})	2262.75	0.5593
Comb(ν_9 ν_{22})	1731.43	0.0002	**ν_{31}**	**2269.74**	**1212.0618**
Comb(ν_{10} ν_{22})	1732.65	0.0923	**Comb(ν_{17} ν_{24})**	**2277.71**	**115.6622**
Comb(ν_{12} ν_{19})	1733.81	0.4094	Comb(ν_{16} ν_{24})	2278.15	0.0002
Comb(ν_{13} ν_{17})	1738.18	0.0040	Comb(ν_{21} ν_{22})	2289.77	23.6029
Comb(ν_9 ν_{23})	1741.83	0.0020	Comb(ν_{21} ν_{23})	2298.47	0.0361
Comb(ν_{10} ν_{23})	1742.16	1.3711	Comb(ν_{13} ν_{28})	2300.77	0.0029
**ν_{31}**	**1745.55**	**158.6465**	Comb(ν_{14} ν_{26})	2304.38	0.0231
Comb(ν_{13} ν_{16})	1745.92	0.1331	Comb(ν_{19} ν_{24})	2307.56	0.1914
Comb(ν_{12} ν_{28})	1748.73	0.0072	Comb(ν_{14} ν_{27})	2308.07	0.0039
**Comb(ν_7 ν_{25})**	**1754.46**	**86.2270**	**Comb(ν_{12} ν_{28})**	**2311.09**	**213.1649**
Comb(ν_{13} ν_{19})	1765.73	0.0264	Comb(ν_{11} ν_{29})	2318.31	0.0001
Comb(ν_4 ν_{27})	1766.51	0.9412	Comb(ν_1 ν_{31})	2320.54	3.3477
Comb(ν_{14} ν_{15})	1767.76	1.7703	Comb(ν_{16} ν_{25})	2322.92	0.0018
Comb(ν_9 ν_{24})	1779.19	0.0029	Comb(ν_{17} ν_{25})	2325.44	0.0474
Comb(ν_7 ν_{26})	1779.70	2.7688	Comb(ν_{10} ν_{30})	2330.61	0.0026
Comb(ν_{10} ν_{24})	1781.77	2.1543	Over(ν_{22})	2333.95	0.0484
Comb(ν_{13} ν_{18})	1785.41	5.1159	Comb(ν_{22} ν_{23})	2341.63	0.2411
Comb(ν_{12} ν_{20})	1795.61	0.6856	Over(ν_{23})	2349.54	0.0307
Comb(ν_4 ν_{28})	1796.27	1.4957	Comb(ν_{19} ν_{25})	2351.97	0.1860
Comb(ν_{11} ν_{21})	1799.16	0.0007	**Comb(ν_2 ν_{31})**	**2353.98**	**52.5362**

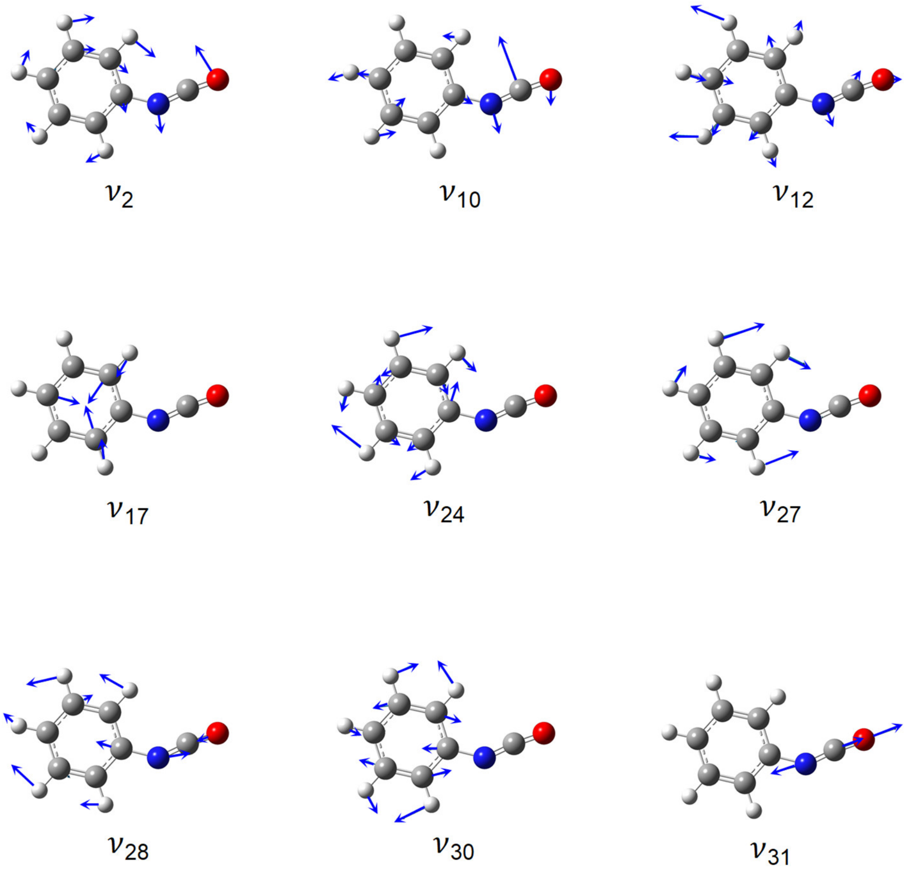

Figure S3. Selected vibration modes of phenyl isocyanate listed in Table S5. These modes were used to assign the experimental spectrum of phenyl isocyanate.

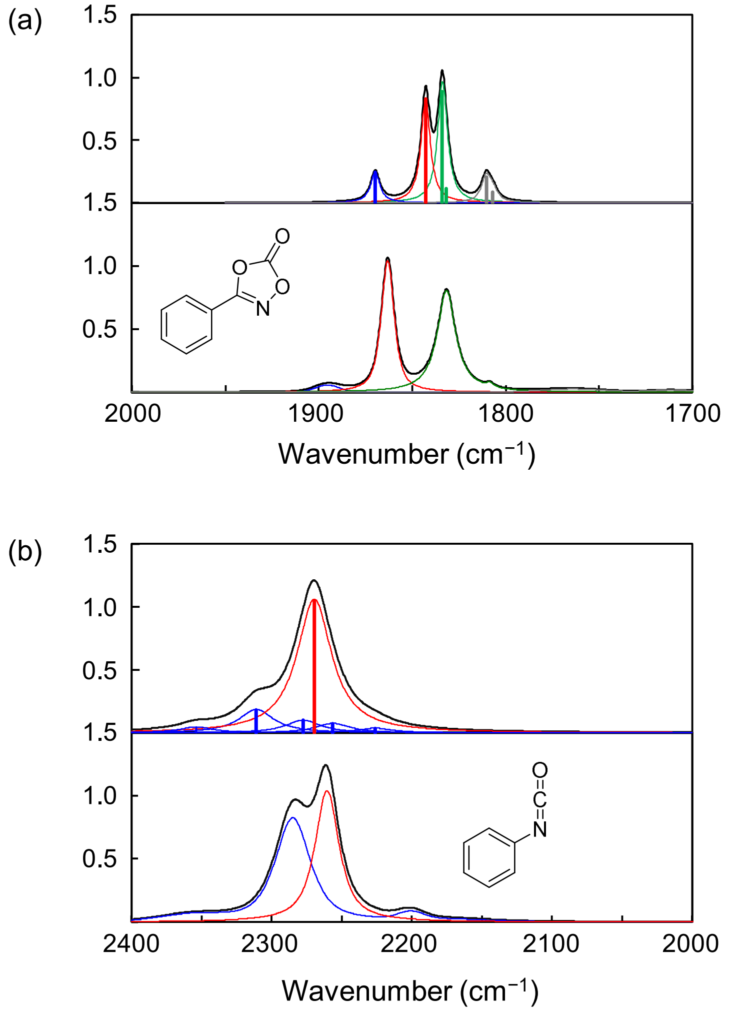

Figure S4. Simulated IR spectra of (a) S_0 3-phenyl-1,4,2-dioxazol-5-one and (b) phenyl isocyanate, together with the corresponding experimental spectra.

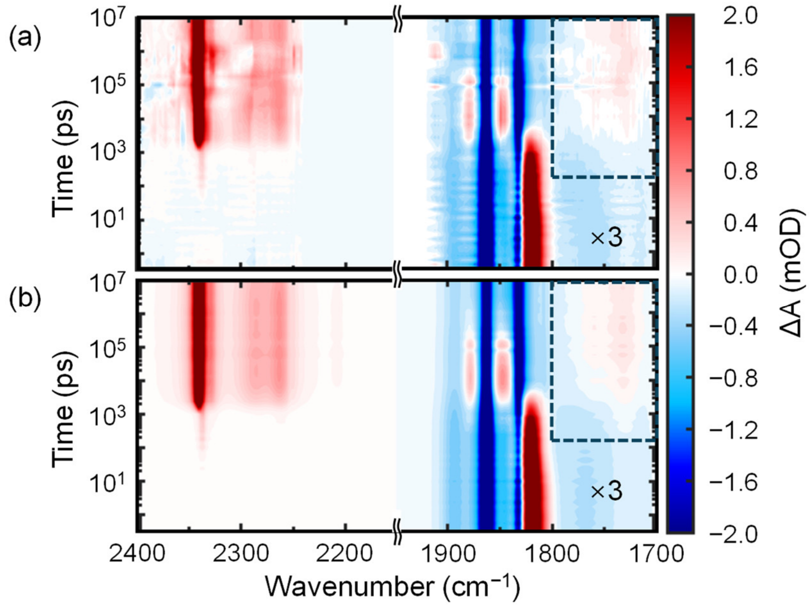

Figure S5. Vertically expanded two-dimensional contour map of (a) the femtosecond time-resolved infrared (TRIR) spectra of 3-phenyl-1,4,2-dioxazol-5-one in $CHCl_3$ at 293 K following 267 nm excitation and (b) the corresponding global fit. To facilitate visualization of weak transient features, the spectra in the 1950–1700 cm^{-1} region are scaled by a factor of 3.
